# Convergent Validity of a Wearable Sensor System for Measuring Sub-Task Performance during the Timed Up-and-Go Test

**DOI:** 10.3390/s17040934

**Published:** 2017-04-23

**Authors:** James Beyea, Chris A. McGibbon, Andrew Sexton, Jeremy Noble, Colleen O’Connell

**Affiliations:** 1 Faculty of Kinesiology, University of New Brunswick, Fredericton, NB E3B5A3, Canada; jbeyea@unb.ca (J.B.); jeremy.noble@unb.ca (J.N.); DrColleenOConnell@horizonnb.ca (C.O.); 2 Institute of Biomedical Engineering, University of New Brunswick, Fredericton, NB E3B5A3, Canada; asexton@unb.ca; 3 Stan Cassidy Centre for Rehabilitation, Fredericton, NB E3BOC7, Canada

**Keywords:** TUG, inertial sensor, motion analysis, activity of daily living, validity, repeatability, minimal detectable change

## Abstract

Background: The timed-up-and-go test (TUG) is one of the most commonly used tests of physical function in clinical practice and for research outcomes. Inertial sensors have been used to parse the TUG test into its composite phases (rising, walking, turning, etc.), but have not validated this approach against an optoelectronic gold-standard, and to our knowledge no studies have published the minimal detectable change of these measurements. Methods: Eleven adults performed the TUG three times each under normal and slow walking conditions, and 3 m and 5 m walking distances, in a 12-camera motion analysis laboratory. An inertial measurement unit (IMU) with tri-axial accelerometers and gyroscopes was worn on the upper-torso. Motion analysis marker data and IMU signals were analyzed separately to identify the six main TUG phases: sit-to-stand, 1st walk, 1st turn, 2nd walk, 2nd turn, and stand-to-sit, and the absolute agreement between two systems analyzed using intra-class correlation (ICC, model 2) analysis. The minimal detectable change (MDC) within subjects was also calculated for each TUG phase. Results: The overall difference between TUG sub-tasks determined using 3D motion capture data and the IMU sensor data was <0.5 s. For all TUG distances and speeds, the absolute agreement was high for total TUG time and walk times (ICC > 0.90), but less for chair activity (ICC range 0.5–0.9) and typically poor for the turn time (ICC < 0.4). MDC values for total TUG time ranged between 2–4 s or 12–22% of the TUG time measurement. MDC of the sub-task times were higher proportionally, being 20–60% of the sub-task duration. Conclusions: We conclude that a commercial IMU can be used for quantifying the TUG phases with accuracy sufficient for clinical applications; however, the MDC when using inertial sensors is not necessarily improved over less sophisticated measurement tools.

## 1. Introduction

The Timed-Up-and-Go (TUG) test is a widely used test for evaluating mobility in patient populations that are frail or have elevated risk of falling [1]. The test is simple to conduct: the patient starts from a seated position, rises, walks a set distance, turns, and walks back to the chair and sits, while an observer measures the total test time with a stop watch [2]. The total TUG time as a measure of physical function is reported to have good reliability and validity [3] in community-dwelling seniors and specific patient populations such as Parkinson’s disease [4], multiple sclerosis [5], stroke [6], and Alzheimer’s disease [7], as well as numerous others. 

However, limiting the measurement of TUG performance to the total time is neglecting a lot of information that might be clinically important. For example, it may be helpful to know if a patient’s poor TUG score is due to slow walking, slow turning, or slow chair activity, or simply due to slower movements for all components of the TUG. Although the idea of quantifying sub-task performance was (to the authors’ knowledge) first proposed and experimentally analyzed in the late 1990s [8], there is still a need to better understand the psychometrics of the TUG’s sub-tasks; recent research suggests that motor deficits undetected from TUG time can be revealed by differences in sub-task performance [9]. 

Several recent studies [10,11,12,13,14,15,16] show that small unobtrusive inertial sensors (inertial measurement units, IMU), are capable of identifying the sub-tasks of the TUG, such as (1) sit-to-stand time; (2) walking time/speed (two walks); (3) turn time/rate (two turns); and (4) stand-to-sit time. Recent studies have explored quantitative TUG analysis in dual-task protocols that are of clinical interest in the study of dementia and falling [16,17]. Furthermore, the technology has created interest in using widely accessible devices that already contain IMU sensors, such as most contemporary smart-phones [18,19,20]. Although these studies show great potential for the added value of quantifying TUG sub-task performance, there are no studies that have rigorously validated TUG phase detection against the industry gold standard in movement analysis—motion capture cameras.

The objectives of this project were: (1) To develop a protocol for acquiring IMU measurements in synchrony with a “gold-standard” optoelectronic motion capture system, during normal and slow TUG tests at two common walking distances (3 m and 5 m) commonly used in the clinic; (2) To validate sub-task performance measures (durations) from IMU sensor data against the sub-task durations from 3D motion analysis data, and; (3) to evaluate the minimal detectable change (MDC) for sensor-based TUG time and sub-task time measurements.

By conducting these experiments using a controlled protocol within a laboratory setting with healthy subjects, we attempt to establish the upper limit on reliability, and lower limit of MDC, of sensor-based sub-task performance measures of the TUG. Furthermore, although MDC values for the total TUG time during conventional testing in clinical research have been reported [21,22,23,24], there are no published data yet on MDC for sensor-based TUG time, or its sub-task times. 

## 2. Methods

### 2.1. Subjects

Twelve healthy adults (height 168 ± 8 cm, and weight 71 ± 14 kg, 6 female) were recruited for this study. Inclusion criteria were being an adult between the ages of 21 and 64 and in good general health. Exclusions were musculoskeletal or neurological conditions affecting gait, uncontrolled hypertension, uncontrolled diabetes, and legal blindness. The study was approved by the institutional ethics review board and all subjects provided informed signed consent prior to participating.

### 2.2. TUG Protocol

A standard TUG protocol was used with the following constraints to maximize experimental control: (1) An armless, backless, and rigid flat seat was used that was height adjusted to knee height (level thigh) with a slightly dorsiflexed (15–20 deg) ankle, greater trochanters approximately 4 cm from the edge of the seat, and feet standing width apart [25]; and (2) The turn-point was measured from a tape line on the floor where the feet rested during sitting, to a tape-cross on the floor either 3 m or 5 m away. Participants were asked to turn when they reached the cross, and not to walk around it.

To ensure that the psychometric properties of sub-task performance measurements reflect the clinical testing environment, we selected two common walking distances used in the clinic: 3 m and 5 m distance between the chair and turning point of the test. In addition, although we recruited healthy volunteers, we asked participants to complete the trials with two different speeds: at their normal speed (cue: “*Please perform the test at your preferred comfortable speed*”), and at slow speed (cue: “*Please perform the test half as fast as normal*”). In addition, each pair of conditions was repeated three times. The order of trials was 3 m normal (3 mN), 5 m normal (5 mN), 3 m slow (3 mS), and 5 m slow (5 mS). A non-random sequence was chosen to ensure that any testing fatigue would preferentially impact the slow trials, rather than being distributed to the normal speed trials.

### 2.3. Motion Measurement

During each TUG trial, participants’ movement kinematics were recorded using two synchronized data acquisition systems: (1) A 12-camera Vicon T160 optoelectronic motion capture system; and (2) A 9-axis Microstrain 3DM-GX1 inertial measurement unit (3-axis accelerometer, 3-axis gyroscope, and 3-axis magnetometer). Synchronization was accomplished by fixing an independent analog 3-axis accelerometer to the 3DM-GX1 case that was directly connected to the Vicon system’s analog-to-digital converter and stored in the output C3D motion data files. 

**Figure 1:**
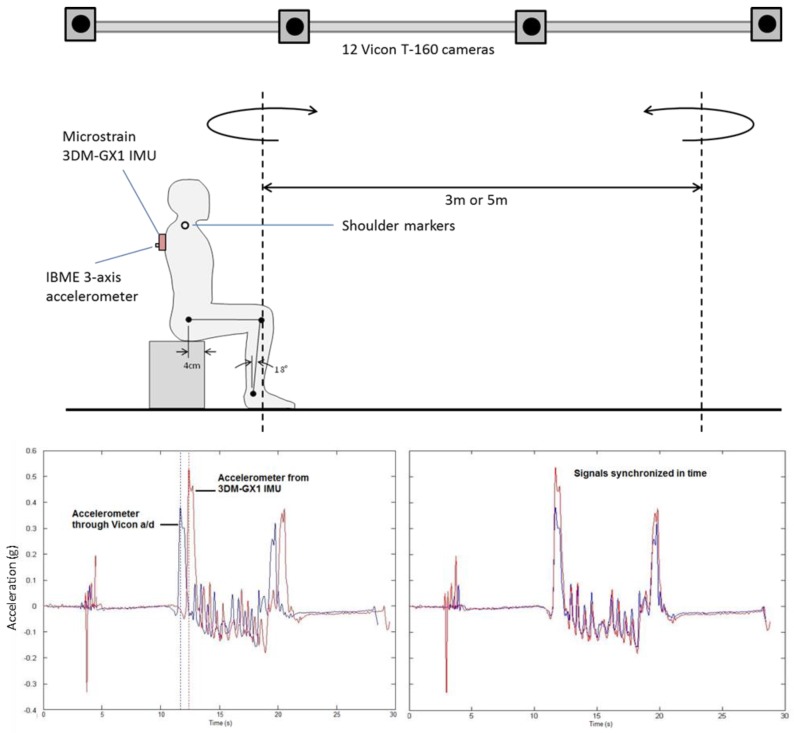
Experimental set-up for the study. Within the viewing volume of the motion capture system (12 Vicon T-160 cameras), the participant performed either a 3 m or 5 m Timed Up and Go (TUG) test, while signals were simultaneously captured with a Microstrain 3DM-GX1 inertial measurement unit (IMU). The IMU produced only a digital output, therefore a separate 3-axis accelerometer was mounted to the 3DM-GX1 for synchronizing the two systems, as shown in the lower portion of the illustration.

Motion capture with the optoelectronic system (sample rate of 100 Hz) followed standard calibration procedures, including a static standing test, conducted prior to and after the TUG session. Preliminary testing indicated that only two reflective markers—on the right and left acromion processes (shoulders)—were required to detect all relevant events of the TUG, as detailed below. The 3DM-GX1 with attached accelerometer (both sampling at 1000 Hz) was securely fixed to the torso of the participant at approximately the T2-T3 spine level (between the shoulder blades), as illustrated in Figure 1. Offline the two data sets were merged and synchronized in time using the anterior-posterior acceleration channel, also shown in Figure 1.

### 2.4. Data Analysis

Two separate heuristic methods were developed, one for the marker data and one for the IMU data, for extracting seven events defined by the following transitions: (1) From quiet sitting to start of sit-to-stand (E1); (2) End of sit-to-stand to start of first walk (E2); (3) End of first walk to start of first turn (E3); (4) End of first turn to start of second walk (E4); End of second walk to start of second turn (E5); End of second turn and start of stand-to-sit (E6); End of stand-to-sit and start of quiet sitting (E7). These event times were then subtracted to measure the total task performance and sub-task performance measures (elapsed time).

#### 2.4.1. TUG Events from Vicon Data

**Figure 2:**
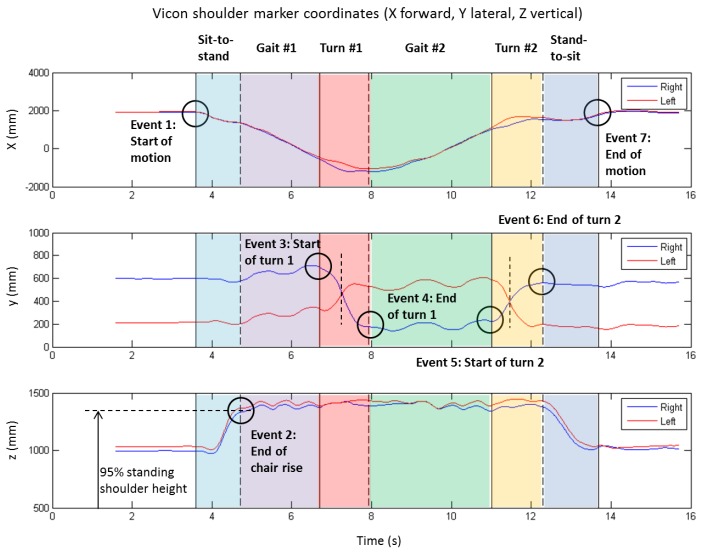
TUG event time determination from motion capture markers on the right and left shoulders. Top: X (anterior/posterior) coordinate is used to detect the start (event 1) and end of movement (event 7). Bottom: Z (superior/inferior) coordinate is then used to detect the end of the sit-to-stand (event 2) when the shoulder marker first exceeds 95% of the standing shoulder height. Middle: The two turns were then detected using the Y (medio/lateral) coordinate, from first locating the cross-point of the left/right shoulder markers (shorted vertical dashed lines), and then locating the maxima and minima before and after (or vice versa depending on turn direction) to define the start (events 3 and 5) and end (events 4 and 6) of the turns.

The algorithm for extracting the above events from the shoulder marker data is illustrated by Figure 2.

First, the right (blue line) and left (red line) shoulder displacements in the anterior-posterior (*X*) direction (Figure 2, top plot) were used to determine the start of movement (E1_V_) and end of movement (E7_V_) events. This was accomplished by filtering the signal (Butterworth low-pass, 6 Hz, 4th order, zero lag), multiplying the first time derivative (velocity) by the second derivative (acceleration), and then using a threshold value (1 × 10^5^ mm^2^/s^3^) to detect the start and end of motion. This approach allowed for some small movements that sometimes occurred at the end of stand-to-sit (e.g., shifting the buttocks, moving the feet, etc.).

Then the two turns were identified using the medio-lateral displacement (*Y*) of the shoulder markers (Figure 2, middle plot). This was accomplished by first locating the cross-over frame of the left and right shoulder trajectories (shown by vertical dashed lines) to mark the mid-turn point, and then searching forward and backward to locate either the first max (or min) in shoulder trajectory prior to (or after) the mid-turn point, or where the X trajectory “gap” closed before and after the turn (see in top plot of Figure 2), whichever happened closest to the mid-turn point. This procedure was employed to find events for the 1st turn (E3_V_ and E4_V_) and 2nd turn (E5_V_ and E6_V_).

Finally the end of stand-to-sit (E2_V_) was located by searching within the window E1_V_–E3_V_ for the first occurrence of average vertical shoulder displacement (*Z*) that exceeds 0.95 of the standing shoulder height (as determined from the static standing trial).

#### 2.4.2. TUG Events from IMU Data

**Figure 3:**
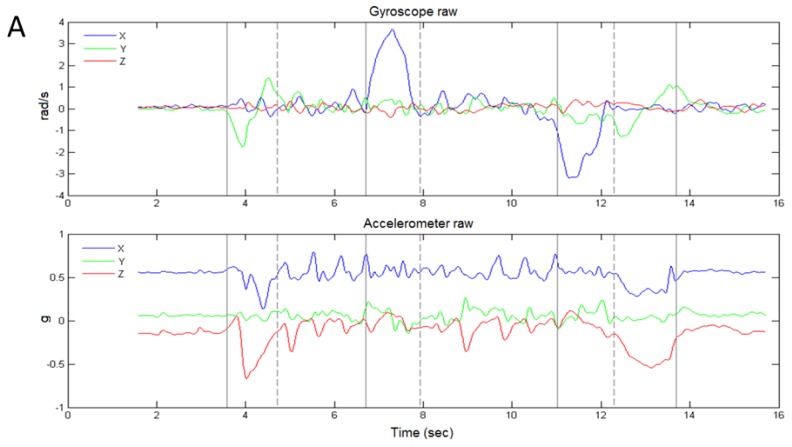

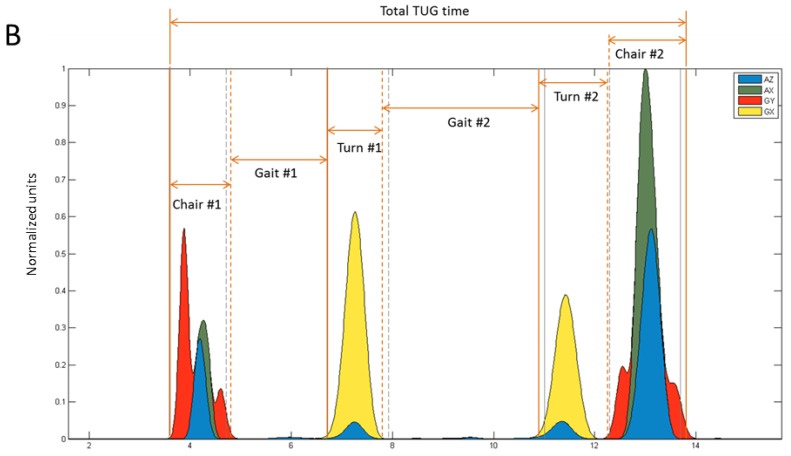
Timed up and go event time determination from the inertial measurement unit’s (IMU) accelerometer and gyroscope channel data. (**A**) When mounted on the torso the IMU’s X axis in the superior/inferior direction, the Y axis is in the medio/lateral direction, and the Z axis is in the anterior/posterior direction. The gyroscope X channel therefore registered the turning motion, and the gyroscope Y and accelerometer Z and X channels registered the chair activity. (**B**) The raw signals were filtered with a zero-lag 4th order Butterworth filter at 10 Hz, rectified, power-scaled, and normalized from 0–1, producing the sensor impulse profile shown in the lower portion of the figure. A fixed threshold value is then used to find the on-off times of each impulse, thus defining the sub-task transition events.

Figure 3 shows an example of sensor data from the IMU during the TUG. Figure 3a shows the raw sensor signals for the gyroscope and accelerometer during the TUG, and Figure 3b shows the conditioned signals from which the sub-task transition events were detected.

Although the accelerometer can be used to detect gait [26], our approach treated gait as a “null signal” such that only the chair activity and turn activity are registered, and thereby indirectly identify the gait sub-tasks of the TUG. Figure 3a shows that turns are registered by the gyroscope *X* channel (*GX*, about vertical axis), and chair activity is registered by the *GY* channel (about the medio-lateral axis), and the accelerometer *X* (*AX*, along vertical axis) and *Z* (*AZ*, along anterior-posterior axis) channels. We therefore used *GX* for detecting the turn events (E3_S_, E4_S_, E5_S_, and E6_S_) and a combination of *GY, AX,* and *AZ* for detecting the chair events (E1_S_, E2_S_, and E7_S_).

Signals were conditioned by first filtering with a Butterworth low-pass filter (10 Hz, 4th order, zero-lag), followed by rectifying and normalizing the signal to its peak value, and then raising the power of the signal to amplify the movement impulse (which remains between 0–1), and then setting a threshold value to find the on-off times. For turns, this approach was used on the *GX* signal, and for chair activity the *GY*, *AX*, and *AZ* signals were first normalized, then summed, and then re-normalized, followed by powering and setting a threshold for on-off detection. The resulting curves (uncombined) and events are depicted in Figure 3b. Power exponents and threshold values used for event detection (on-off times) are shown in Appendix A.

### 2.5. Statistical Analysis

Statistical analyses were conducted using SPSS (v21, IBM Corp.) and Matlab (Mathworks Inc. Natick, MA, USA).

#### 2.5.1. Validity of Sub-Task Performance Measures

Data were analyzed separately for each of the four conditions tested: 3 m normal, 5 m normal, 3 m slow, and 5 m slow. First, the six sub-task (or phases, P) and total TUG times were computed for sensor-based {P1_S_ = E2_S_ − E1_S_; P2_S_ = E3_S_ − E2_S_; …; P6_S_ = E7_S_ − E6_S_; P7_S_ = E7_S_ − E1_S_} and marker-based {P1_V_ = E2_V_ − E1_V_; P2_V_ = E3_V_ − E2_V_; …; P6_V_ = E7_V_ − E6_V_; P7_V_ = E7_V_ − E1_V_} systems and averaged across the *k* = 3 repetition trials. Relative error was calculated as the mean of the differences in paired data {P1_S_, P1_V_; P2_S_, P2_V_; …; P7_S_, P7_V_} and was tested against a mean difference of zero using the 2-tailed paired-samples *t*-tests (95% confidence interval, CI).

Intra-Class Correlation (ICC) analysis (mixed 2-way ANOVA for absolute agreement between *k* means [27], commonly called the ICC(2,k) model) was then used to quantify the agreement between the sensor-based and marker-based sub-task performance measures. To differentiate this ICC from the others below, we will refer to this as the between-methods ICC, or ICC_b_. Typically ICC values above 0.7 are taken to represent acceptable agreement, between 0.7 and 0.5 as poor agreement, and less than 0.5 as no agreement [28]. In addition, we computed the 95% CI on the ICC_b_ values, where the CI boundaries that enclose zero are non-significant (agreement level is not different from zero).

#### 2.5.2. Minimal Detectable Change

The minimal detectable change (95% confidence MDC^95^) in performance for each sub-task, and the total task, was evaluated for repeated sensor-based measures and also for repeated marker-based measures. The MDC was computed from the standard error of measurement (SEM)

$${\mathrm{MDC}}^{95}=\mathrm{SEM}\times \sqrt{2}\times 1.96$$

where 1.96 is the z-score for 95% confidence, and SEM was computed from the within-subjects variance, as reflected by the ICC_w_ of trial repetitions.

$$\mathrm{SEM}={\mathrm{SD}}_{w}\times \sqrt{1-{\mathrm{ICC}}_{w}}$$

where SD_w_ is the within-subjects variance.

Finally we computed the ratio of MDC^95^ to the corresponding mean duration in order to evaluate the impact of the MDC on the measurement requirement.

## 3. Results

Of the twelve subjects that participated, data for one participant was completely excluded due to technical failure of the IMU. For the slow 5 m trials, there was one participant whose shoulder markers went outside the camera’s viewing volume for all of their trial repetitions. The data below therefore reflect *n* = 10 for the 5 m slow trials, and *n* = 11 for the other three conditions. 

### 3.1. Validation of Sub-Task Performance Measures

Convergent validity was assessed by evaluating the relative error between the gold standard and the sensor-based measurements, and by evaluating the absolute agreement between the gold standard and the sensor-based measurements. These data can be viewed in Appendix A, Table A1.

#### 3.1.1. Relative Error

**Figure 4:**
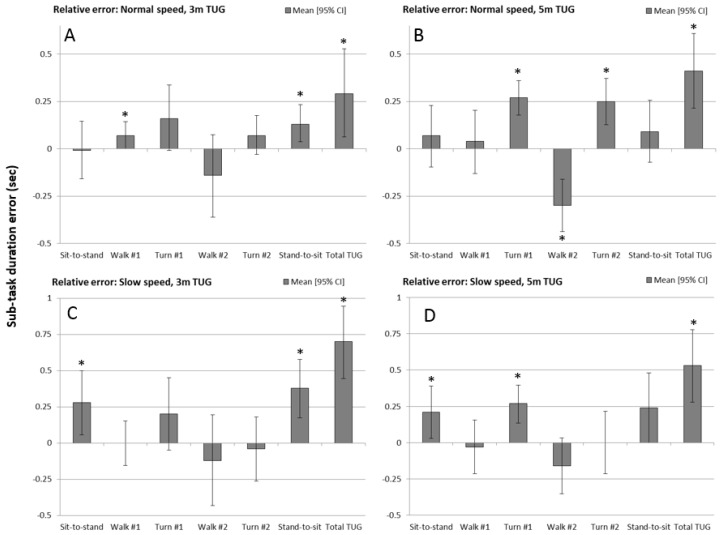
Relative error between the marker-based and IMU-based measurement systems for sub-task phases and the total TUG time, for each of the four experimental conditions: (**A**) 3 m normal speed; (**B**) 5 m normal speed; (**C**) 3 m slow speed, and; (**D**) 5 m slow speed. Whiskers represent the 95% confidence intervals on the mean error and the asterisk indicates error that was significantly different from zero (*p* < 0.05).

The mean difference in sub-task times between the 3D motion capture marker data (‘gold standard’) and IMU sensor, for each of the experimental test conditions, is shown by the bar charts in Figure 4 (also see Table A1). Positive values indicate that the sensor measured values lower than the gold-standard. Bar charts include 95% confidence intervals (whiskers) and the asterisks indicate which sub-task duration measures were significantly different from zero. 

The total TUG time (E7 minus E1) error was consistently significant at *p* < 0.05 on a paired-samples *t*-test. Although the pattern of relative error was consistent across experimental conditions for the sub-tasks, errors were more sporadic between the four experimental conditions. For the 3 m normal speed TUG, the 1st walk and stand-to-sit duration error were both significantly different from zero (under-predicted by the sensor). For the 5 m normal speed TUG, both turns (under-predicted) and the 2nd walk (over-predicted) had errors greater than zero. For the 3 m slow speed TUG, the sit-to-stand and stand-to-sit duration errors (under-predicted) were significant, and for the 5 m slow speed TUG, the sit-to-stand and 1st turn duration errors (under-predicted) were significantly different from zero. 

It is worth noting that the error magnitude for sub-task durations fell mostly in the region of ±0.25 s for all four experimental conditions. For the total TUG time, the mean error between the marker-based and sensor-based measurements fell between 0.25 s and 0.75 s, with the slow speed condition being at the higher end of this range. 

#### 3.1.2. Absolute Agreement

**Figure 5:**
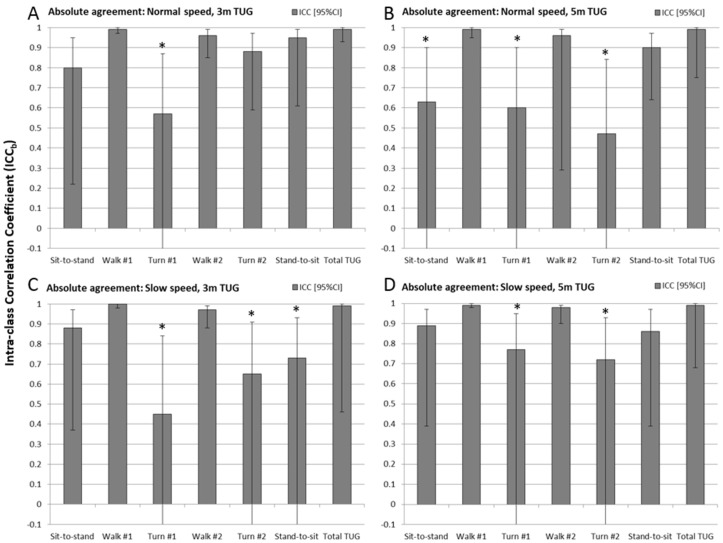
Intraclass correlation between measures (ICC_b_) showing the level of absolute agreement and confidence of that agreement level, for each sub-task phase and total timed up and go time, for each of the four experimental conditions: (**A**) 3 m normal speed; (**B**) 5 m normal speed; (**C**) 3 m slow speed, and; (**D**) 5 m slow speed. Whiskers represent the 95% confidence intervals on the ICC and the asterisk indicates sub-tasks where the 95% confidence interval enclosed zero, and was therefore non-significant (*p* > 0.05).

Results of the Intra-class Correlation Coefficient analysis between methods (ICC_b_) are shown for each experimental condition in the bar charts of Figure 5 (also see Table A1). The whiskers represent 95% confidence intervals, which for the ICCs are not symmetric above and below the ICC_b_ value (since the ICC range limit is 1 to −1). The ICC_b_s with confidence intervals including zero are marked with an asterisk.

Total TUG time and both walk times were found to have excellent absolute agreement (ICC_b_ > 0.95) for all four experimental conditions. For chair activities the ICC_b_ values ranged between 0.80 and 0.95 for three of the four experimental conditions, but lower (0.6) and non-significant (CI included zero) for the 5 m TUG at normal speed. Turns were found to be in poor to no agreement with all but one instance (turn #2 for 3 m normal speed TUG, ICC_b_ = 0.88) having very low ICC_b_ values (0.4–0.75) with CI boundaries that include zero. 

### 3.2. Repeatability and MDC of Sub-Task Performance Measures

Analysis of within-subjects effects were conducted to evaluate the repeatability of both the gold standard (given that it is not a true ‘gold standard’) and the sensor-based measurements, and to evaluate the MDC (95%) of both measurement systems. These data can be viewed in Appendix A, Table A2.

#### 3.2.1. Repeatability of Sub-Task Measures

**Figure 6:**
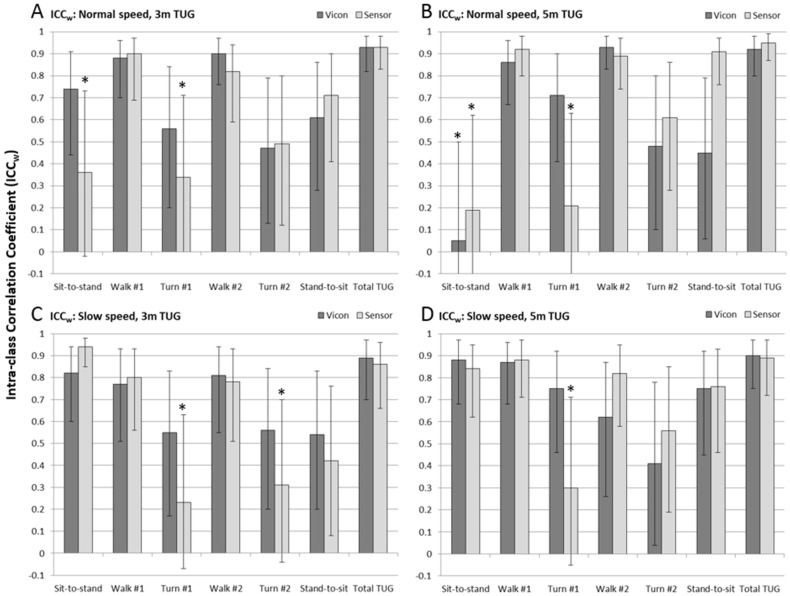
Intraclass correlation within measures (ICC_w_) showing the level of repeatability of sensor (light gray) and Vicon (dark gray) measurements of sub-task performance, for each of the four experimental conditions: (**A**) 3 m normal speed; (**B**) 5 m normal speed; (**C**) 3 m slow speed, and; (**D**) 5 m slow speed. Whiskers represent the 95% confidence intervals on the ICC and the asterisk indicates sub-tasks where the 95% confidence interval enclosed zero, and was therefore non-significant (*p* > 0.05).

Results of the Intra-class Correlation Coefficient analysis performed on subjects’ repeated trials within the experimental conditions (ICC_w_) are shown in Figure 6 (see also Table A2) for measurements from the marker-based analysis (Vicon, dark gray bars) and the IMU-based analysis (Sensor, light gray bars). As above, whiskers represent the 95% confidence interval, and the asterisk represents the ICC_w_ results that were non-significant (CI encloses zero).

Overall it can be seen that repeatability was higher (and with higher confidence) for the gold standard Vicon measurements than for the IMU-based measurements. A notable exception was the sit-to-stand sub-task of the 5 m normal speed TUG test, which had lower than normal repeatability. In all other circumstances, however, it was only the sensor result that showed lack of confidence in repeatability, primarily of the measurement of the 1st turn time (all four experimental conditions), the sit-to-stand time (normal speed trials of both distances), and the 2nd turn (3 m slow speed). 

#### 3.2.2. Minimal Detectable Change

**Figure 7:**
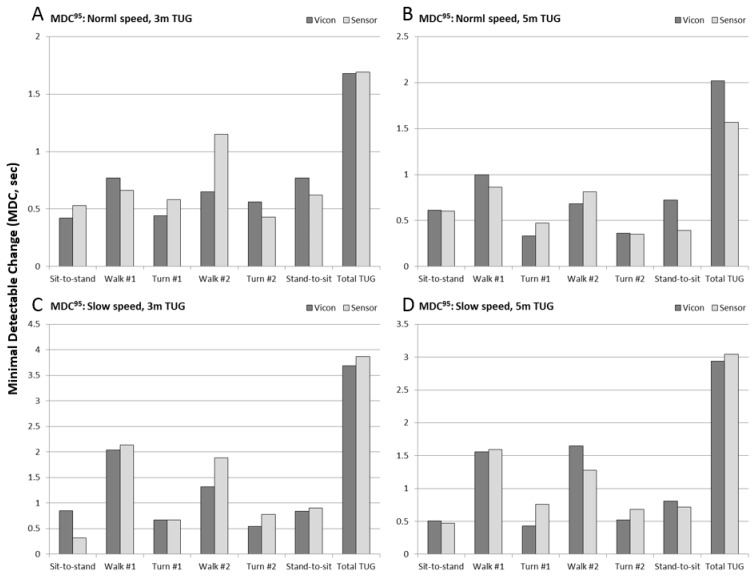
Minimal detectable change at 95% confidence (MDC^95^) for sub-task and total TUG performance measures for the sensor (light gray) and Vicon (dark gray) measures, for each of the four experimental conditions: (**A**) 3 m normal speed; (**B**) 5 m normal speed; (**C**) 3 m slow speed, and; (**D**) 5 m slow speed.

Results of 95% Minimal Detectable Change (MDC^95^) are shown in Figure 7 (see also Table A2) for the six sub-task durations and total TUG time, for each of the four experimental conditions. MDC values for marker-based measures (Vicon) are shown in dark gray, and IMU-based measures (Sensor) are shown in light gray. For normal speed trials, the MDC values for turns and chair rise were approximately 0.5 s, between 0.5 and 1.0 s for walk times, and 1.5–2 s for total TUG time. For slow speed trials, the turn and chair rise MDC remained the same, whereas the walk time MDC increased to 1.5–2 s, and the total TUG time MDC increased to 3–4 s.

**Figure 8:**
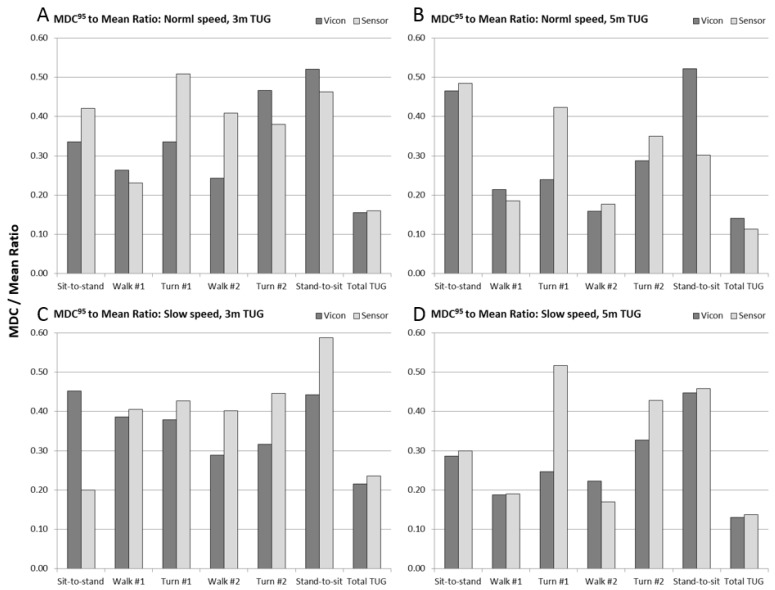
Ratio of minimal detectable change at 95% confidence (MDC^95^) to mean duration of the sub-task and total timed up and go performance measures for the sensor (light gray) and Vicon (dark gray) measures, for each of the four experimental conditions: (**A**) 3 m normal speed; (**B**) 5 m normal speed; (**C**) 3 m slow speed, and; (**D**) 5 m slow speed.

To put the MDC^95^ values into the context of their measurement requirements, we finally computed the ratio of MDC^95^ to the mean duration of the sub-task. These data are shown in Figure 8 (see also Table A2). For the total TUG time, the MDC was between 12–22% of the measured magnitude of the test time. For walking sub-tasks, the MDC was between 18–40% of the measured walk times, and for turns and chair rise, the MDC was between 20–60% of the measured sub-task duration. 

## 4. Discussion

The TUG test is one of the most common timed tests of physical function used in clinical research and practice, owing greatly to its relative simplicity. The test can be completed in a few minutes and all one needs is a chair, a tape measure and a stop watch. The total TUG time has been widely studied in numerous patient populations, and shown to be a valid measure of physical function [1,3,4,5,6,7] and its minimal detectable change in various populations has been established [21,22,23,24]. However, in its current form it has no diagnostic capability. The total TUG time is simply a composite measure of the patient’s performance on the various sub-tasks of rising from and sitting in a chair, turning, and walking. 

Several groups have explored wearable sensors—IMUs specifically—as a potential approach for enabling the quantification of sub-task performance measures of the TUG and have demonstrated that these sensor-based metrics can discriminate between healthy controls and patient populations [10,11,12,13,14,15,20]. These devices have been shown to be useful for assessments in community dwelling seniors with dementia and/or history of falling when incorporated within a clinical protocol, such as a single- and dual-task paradigm [16,17,29]. Although not meant to be an exhaustive review, we discuss below some recent advances using wearable sensors during the TUG test, and the specific contributions of the present work to furthering this base of knowledge.

### 4.1. IMU-Based Measurement of TUG Sub-Tasks

The concept of the “iTUG” was introduced by Salarian et al. [13], who compared patients with Parkinson’s disease (PD) to healthy controls using an instrumented TUG test (with commercial IMUs positioned on the shank and sternum) parsed into four phases: Sit-to-stand, Gait, Turning, and Turn-to-sit. It was found that the resulting phases could be used to determine if the subject has movement symptoms of PD, whereas the total TUG time was not able to discriminate between groups. A study by Reinfelder et al. [12] also examined TUG phase segmentation in patients with PD, parsing the TUG into five phases: Sit to walk, Walking, First turn, Second turn, and Turn to sit. This study evaluated a variety of classifiers for segmenting the TUG into its sub-task phases, and found that a support vector machine performed best for correctly classifying PD and healthy controls (82%).

Weiss et al. [11] used accelerometry to analyze the sit-to-stand and stand-to-sit portions of the TUG in community dwelling fallers and non-fallers and found the instrumented TUG test correctly classified faller and non-fallers with an accuracy of 87%, whereas the standard clinical stop watch approach yielded only a 63% classification accuracy. Fall-risk assessment with an instrumented 3 m TUG test has also been studied by Zakaria et al. [14] and Tmaura et al. [10]. The TUG was parsed into eight phases; the six we describe except the sit-to-stand and stand-to-sit phases were each divided into two sub-phases delineated by the trunk bend. These studies showed that elderly high fall-risk and low fall-risk groups could be discriminated from healthy subjects. 

Finding from these studies are in good agreement with our “normal” and “slow” speed trial results, as shown in Table 1, and suggests that our data has a similar range to that of the clinical studies. Comparison with other published studies is difficult either due to the test being a different distance (7 m or 10 m) or because the sub-task definitions are too dissimilar. The fact that our healthy subjects normal speed was similar to Zakaria’s healthy (low fall-risk) seniors, rather than faster (as one might expect), is probably explained by the fact that their patient participants were instructed to walk as fast as possible, whereas our healthy participants were asked to walk at their preferred speed. Furthermore, although we asked our participants to walk at half their normal speed, they actually choose a slow speed that was approximately 2/3 of their normal speed and resulted in a slightly longer TUG test than recorded for the seniors with high-fall risk in Tmaura’s study.

### 4.2. Psychometric Properties of the Instrumented TUG 

A series of recent studies by Galan-Marcant et al. [20,30,31] have explored using the IMU in a smart phone to parse a 10 m TUG test into five phases: Sit-to-stand, Gait, Turning, Returning gait, and Turn-to-sit, when mounted on the subject’s sternum, and showed it was possible to distinguish frail from non-frail elderly when comparing groups by phase durations that could not be discriminated based on the total TUG time [31]. Measurements from the phone’s IMU were validated against a commercial IMU (minima and maxima accelerations rather than time durations) and showed a high level of agreement between sensors with ICC > 0.8, and high similarity (>0.8) of signal waveforms during sub-task measurements using the Coefficient of Multiple Correlation analysis (CMC). They did not evaluate the agreement in sub-task performance measures, however, so these results cannot be directly compared to ours. 

Wuest et al. [15] found good test-retest reliability of IMU-based TUG metrics in patients with stroke, with ICC values generally above 0.9 for TUG sub-tasks with the exception of sit-to-walk (equivalent to our sit-to-stand sub-task) which had a poor reliability (0.4). This finding agreed with our data to some extent, as it was the longer 5 m test that experienced the lower sit-to-stand reliability in our study, and Wuest’s study used a 7 m TUG test. In disagreement was that their ICCs for turn duration were very good (>0.90), whereas our data showed relatively low ICC_w_ values for turns (<0.4). However, when viewing the current study’s data in Figure 4, it is clear that the error range was relatively small. Given that all participants in the current study were healthy, it is not surprising that the variability for turn and chair activity times were small (approx. 1/4 s). Because ICC analysis is known to suffer from a compressed range of values [28], this may have contributed to the low values we found. Others have also reported lower reliability of the turning portion of the TUG [32].

Smith et al. [16] recently studied the test-retest reliability of the quantitative TUG during single and dual (motor and cognitive) task conditions using a commercial system (Kinesis QTUG™) tailored for this application. Although some measures, such as total TUG time and walking times had acceptable reliability (ICC > 0.7), similar to our findings the reliability of the turn metrics were found to be low (ICC < 0.5). Furthermore, they found the variability increased when adding the dual task, lowering the reliability. These studies reinforce why it is critically important to analyze the psychometric properties of IMU-based clinical measures.

Although others have not published MDC for the instrumented TUG sub-task performance measures, there is a relative abundance of published MDC values for the clinical TUG test in a variety of populations [21,22,23,24]. What is striking is that the MDC we computed for the total TUG time, for both the sensor-based measurement as well as the marker-based “gold-standard” measurement, ranged between 1.5–2 s for normal speed trials and 3–4 s for slow speed trials. This is in excellent agreement with the published literature on the MDC of the clinical TUG test when using a stop watch, but also suggests that the variability within subjects repeating the test is a greater source of error than the measurement technique itself. The further implication is that adopting IMU-based measurement systems for timed tests of physical function may not necessarily improve our ability to detect meaningful change in the test’s output metrics. 

For the sub-task MDC to mean ratio (Figure 8 and Table A2), it can be seen that for chair activity and turns in particular, the calculated MDC is between 1/4 and more than half of the magnitude of the sub-task duration. The implication of this finding is that individuals’ sub-task performance measures would need to change by a considerable amount in order to be reliably detected with IMU sensors. Only moderately better results were obtained with a state-of-the-art motion capture system, which suggests that our results expose the fundamental limitations of fidelity in quantifying sub-task performance with wearable sensors.

## 5. Conclusions

Establishing how well we can expect IMU technology to perform in the clinic has in the past only been studied from a concurrent validity perspective—several studies show that the instrumented TUG can discriminate between patient groups that ought to differ. Our study examined the convergent validity of IMU technology with an accepted gold-standard measurement technology in the field, and therefore fills an important gap by establishing the upper-bound of measurement fidelity of an IMU-based TUG sub-task measurement system that is independent of the patient population.

Although absolute agreement using ICC analysis between measurement systems was high for the total TUG time and walk times, the relative error analysis showed there was a tendency to under-predict the total TUG time and over-predict the 2nd walk time, although only the former was found to be statistically significant. Absolute agreement, however, was not established for the turn duration measurements and questionably so for the chair activity duration measurements. Nevertheless, the relative errors were small and suggest that the compressed variability of this sub-task in a healthy population may have contributed to the low ICC values.

Analysis of measurement repeatability and the ability to detect a statistically meaningful change largely reflect the results of the comparison between measurement methods. Total TUG time and the walk times were highly repeatable (ICC > 0.9), with chair activities and turns being less so and with a considerable range across the experimental conditions. MDC values for the sensor-based TUG measurement were equivalent to published values from clinical experiments using standard stop watch technology, which suggests that the largest source of error in the TUG test is the variability within subjects rather than the measurement approach.

## 6. Limitations

There are several limitations to our study that must be addressed. The sample size was relatively small (*n* = 12) though similar to healthy control sample sizes reported by others [13,16,20]. More importantly we did not study a patient or senior population. There were two reasons for this: (1) the protocol was rather lengthy with the repeated trials and four experimental conditions, which may be challenging for a patient population, and (2) our intent was to validate the measurement instrument independent of a patient population. In order to capture a range of realistic data, however, we had our healthy participants perform the test at an artificially slower speed. Although participants did walk at approximately 2/3 of their preferred speed, it is likely they performed the turns and chair activities closer to their normal speed. 

Another related issue is that the TUG test instruction according to Podsiadlo and Richardson [2] has patients perform the test as fast (and safe) as they can. Our participants were asked, however, to walk at their preferred “normal” speed. In pilot testing the protocol it was discovered that asking healthy participants to complete the TUG as fast as possible resulted in a very different test. For this reason, only preferred speed and slow speed trials were performed by the study sample. Comparison with a senior and high fall-risk population sample in Table 1 suggests that we did in fact capture the relevant range of TUG results for a clinical population.

We only studied distances of 3 m and 5 m, whereas a number of instrumented TUG tests use the extended TUG, at either 7 m or 10 m. There were two reasons for our selection: (1) in reviewing the literature the clinical TUG test is almost always a 3 m test, and therefore we wanted psychometric properties of the test similar to that done clinically; and (2) the viewing volume of the Vicon motion capture system was put to its limits in order to capture the full 5 m test, and thus represented the longest test we could capture from start to finish in the motion capture lab.

The testing sequence was not randomized. The reason for this was also twofold: (1) capturing the 3 m and 5 m tests required modification to the start and stop points of the test, which meant they could not be feasibly randomized among different speeds; and (2) the speed factor also could not be feasibly randomized as it was not a reasonable expectation for healthy participants to mimic slow walking repeatedly if having to do it at random times. The sequence selected: 3 m tests (normal then slow) then 5 m tests (normal and slow), was therefore implemented to reduce experimental and participant burden and to allow the slow trials to capture any testing fatigue during the 3 m and 5 m test batteries.

Finally, we only studied one approach for segmenting the IMU data into the relevant phases of the TUG. Several approaches have been published, ranging from threshold values in real physical units (m/s^2^ or g’s) to machine learning algorithms for classifying motion segments into sub-task categories. We wanted to avoid using thresholds based on real physical values, as this can be problematic due to alignment issues of the sensor with major motion axes or calibration differences between different IMU sensors. On the other hand, machine learning and other numerical approaches to segmentation require training, and we wanted to avoid having the limitation of the training with different populations. As such, the algorithm employed in this study is a relatively simple signal conditioning procedure that does not rely on real physical units for establishing thresholds or search windows, but rather exploits the impulsive behavior of IMU sensor channels during the TUG. Combining this with the known degree-of-freedom of IMU channels (what movements they can measure) enables identification of the start and end of a movement segment, such as a turn or chair rise stand. Because it is easy to implement, we believe the algorithm described here may also be applicable for quantifying movement segments or phases of other clinical tests of physical function.

## Figures and Tables

**Table 1:** Comparison of normal and slow TUG sub-task times with other studies.

	Sub-Task Durations (s)—mean ± 1 s.d.			
Source	This study	Zakaria et al. *	This study	Tmaura et al. *
Subjects	Young adult subjects, normal speed	Healthy senior, low fall-risk	Young adult subjects, slow speed	Seniors with high fall-risk
Sit-to-stand	1.25 ± 0.30	1.44 ± 0.36	1.88 ± 0.73	1.67 ± 0.55
Walk #1	2.92 ± 0.80	2.21 ± 0.67	5.28 ± 1.54	4.05 ± 1.10
Turn #1	1.31 ± 0.24	1.61 ± 0.48	1.77 ± 0.36	2.50 ± 0.65
Walk #2	2.76 ± 0.75	2.41 ± 0.67	4.58 ± 1.10	3.85 ± 0.67
Turn #2	1.20 ± 0.28	1.18 ± 0.37	1.71 ± 0.30	1.80 ± 0.19
Stand-to-sit	1.48 ± 0.45	1.95 ± 0.50	1.90 ± 0.45	2.89 ± 0.57
Total TUG	10.82 ± 2.33	10.8 ± 1.28	17.13 ± 3.94	15.81 ± 1.66

* Chair times are the combined times of chair sub-phases.

## Abbreviations

The following abbreviations are used in this manuscript:

TUG	Timed Up-and-Go
IMU	Inertial Measurement Unit
ICC	Interclass Correlation Coefficient
SEM	Standard Error of Measurement
MDC	Minimal Detectable Change

## Appendix A

**Table A1:** Table of measurements for marker-based (Vicon) and IMU-based (Sensor) TUG sub-tasks, as well as the relative error and absolute agreement (ICC_b_).

TUG Sub-Task	Vicon		Sensor		Rel. Error		Sig.	ICC [95% CI]		
	Mean	SD	Mean	SD	Mean	SD	p	ICCb	Low	Upp
Speed = Normal; Distance = 3 m (*n* = 11)										
Sit-to-stand	1.25	0.30	1.26	0.24	−0.01	0.23	0.927	0.80	0.22	0.95
Walk #1	2.92	0.80	2.85	0.75	0.07	0.11	0.049	0.99	0.97	1.00
Turn #1	1.31	0.24	1.14	0.26	0.16	0.26	0.060	0.57	−0.27	0.87
Walk #2	2.67	0.75	2.81	0.96	−0.14	0.32	0.171	0.96	0.85	0.99
Turn #2	1.20	0.28	1.13	0.22	0.07	0.15	0.140	0.88	0.59	0.97
Stand-to-sit	1.48	0.45	1.34	0.41	0.13	0.14	0.011	0.95	0.61	0.99
Total TUG	10.82	2.33	10.53	2.35	0.29	0.35	0.018	0.99	0.93	1.00
Speed = Normal; Distance = 5 m (*n* = 11)										
Sit-to-stand	1.31	0.22	1.24	0.24	0.07	0.24	0.389	0.63	−0.34	0.90
Walk #1	4.68	0.96	4.65	1.09	0.04	0.25	0.649	0.99	0.95	1.00
Turn #1	1.38	0.22	1.11	0.19	0.27	0.14	0.000	0.60	−0.20	0.90
Walk #2	4.28	0.95	4.58	0.90	−0.30	0.21	0.001	0.96	0.29	0.99
Turn #2	1.25	0.18	1.00	0.20	0.25	0.18	0.001	0.47	−0.29	0.84
Stand-to-sit	1.38	0.35	1.29	0.45	0.09	0.24	0.241	0.90	0.64	0.97
Total TUG	14.28	2.63	13.87	2.67	0.41	0.29	0.001	0.99	0.75	1.00
Speed = Slow; Distance = 3 m (*n* = 11)										
Sit-to-stand	1.88	0.73	1.60	0.48	0.28	0.33	0.019	0.88	0.37	0.97
Walk #1	5.28	1.54	5.28	1.73	0.00	0.23	0.986	1.00	0.98	1.00
Turn #1	1.77	0.36	1.57	0.28	0.20	0.37	0.103	0.45	−0.57	0.84
Walk #2	4.58	1.10	4.70	1.45	−0.12	0.47	0.420	0.97	0.88	0.99
Turn #2	1.71	0.30	1.75	0.34	−0.04	0.33	0.694	0.65	−0.41	0.91
Stand-to-sit	1.90	0.45	1.53	0.43	0.38	0.30	0.002	0.73	−0.21	0.93
Total TUG	17.13	3.94	16.44	3.79	0.70	0.37	0.000	0.99	0.46	1.00
Speed = Slow; Distance = 5 m (*n* = 10)										
Sit-to-stand	1.78	0.54	1.57	0.42	0.21	0.25	0.027	0.89	0.39	0.97
Walk #1	8.33	1.57	8.36	1.68	−0.03	0.26	0.726	0.99	0.98	1.00
Turn #1	1.74	0.31	1.47	0.33	0.27	0.18	0.001	0.77	−0.22	0.95
Walk #2	7.40	0.96	7.56	1.08	−0.16	0.27	0.093	0.98	0.90	0.99
Turn #2	1.59	0.24	1.59	0.37	0.00	0.30	0.981	0.72	−0.23	0.93
Stand-to-sit	1.81	0.58	1.57	0.52	0.24	0.34	0.052	0.86	0.39	0.97
Total TUG	22.64	3.37	22.11	3.25	0.53	0.35	0.001	0.99	0.68	1.00

**Table A2:** Repeatability and minimal detectable change for marker-based (Vicon) and IMU-based (Sensor) sub-task measurements.

TUG Sub-Task	Vicon					Sensor				
	ICCw	Low	Upp	MDC (s)	MDC Ratio	ICCw	Upp	Low	MDC (s)	MDC Ratio
Speed = Normal; Distance = 3 m (*n* = 11)										
Sit-to-stand	0.74	0.44	0.91	0.42	0.34	0.36	−0.02	0.73	0.53	0.42
Walk #1	0.88	0.70	0.96	0.77	0.26	0.90	0.69	0.97	0.66	0.23
Turn #1	0.56	0.20	0.84	0.44	0.34	0.34	0.00	0.71	0.58	0.51
Walk #2	0.90	0.76	0.97	0.65	0.24	0.82	0.59	0.94	1.15	0.41
Turn #2	0.47	0.13	0.79	0.56	0.47	0.49	0.12	0.80	0.43	0.38
Stand-to-sit	0.61	0.28	0.86	0.77	0.52	0.71	0.41	0.90	0.62	0.46
Total TUG	0.93	0.82	0.98	1.68	0.16	0.93	0.83	0.98	1.69	0.16
Speed = Normal; Distance = 5 m (*n* = 11)										
Sit-to-stand	0.05	−0.24	0.50	0.61	0.47	0.19	−0.16	0.62	0.60	0.48
Walk #1	0.86	0.67	0.96	1.00	0.21	0.92	0.80	0.98	0.86	0.18
Turn #1	0.71	0.41	0.90	0.33	0.24	0.21	−0.11	0.63	0.47	0.42
Walk #2	0.93	0.83	0.98	0.68	0.16	0.89	0.74	0.97	0.81	0.18
Turn #2	0.48	0.10	0.80	0.36	0.29	0.61	0.28	0.86	0.35	0.35
Stand-to-sit	0.45	0.06	0.79	0.72	0.52	0.91	0.76	0.97	0.39	0.30
Total TUG	0.92	0.80	0.98	2.02	0.14	0.95	0.87	0.99	1.57	0.11
Speed = Slow; Distance = 3 m (*n* = 11)										
Sit-to-stand	0.82	0.60	0.94	0.85	0.45	0.94	0.85	0.98	0.32	0.20
Walk #1	0.77	0.51	0.93	2.04	0.39	0.80	0.56	0.93	2.14	0.41
Turn #1	0.55	0.17	0.83	0.67	0.38	0.23	−0.07	0.63	0.67	0.43
Walk #2	0.81	0.55	0.94	1.32	0.29	0.78	0.51	0.93	1.89	0.40
Turn #2	0.56	0.20	0.84	0.54	0.32	0.31	−0.04	0.70	0.78	0.45
Stand-to-sit	0.54	0.20	0.83	0.84	0.44	0.42	0.08	0.76	0.90	0.59
Total TUG	0.89	0.70	0.97	3.69	0.22	0.86	0.66	0.96	3.87	0.24
Speed = Slow; Distance = 5 m (*n* = 10)										
Sit-to-stand	0.88	0.68	0.97	0.51	0.29	0.84	0.62	0.95	0.47	0.30
Walk #1	0.87	0.68	0.96	1.56	0.19	0.88	0.71	0.97	1.59	0.19
Turn #1	0.75	0.46	0.92	0.43	0.25	0.30	−0.05	0.71	0.76	0.52
Walk #2	0.62	0.26	0.87	1.65	0.22	0.82	0.58	0.95	1.28	0.17
Turn #2	0.41	0.04	0.78	0.52	0.33	0.56	0.19	0.85	0.68	0.43
Stand-to-sit	0.75	0.45	0.92	0.81	0.45	0.76	0.46	0.93	0.72	0.46
Total TUG	0.90	0.75	0.97	2.94	0.13	0.89	0.72	0.97	3.04	0.14
